# The St. Louis Dental Society

**Published:** 1873-04

**Authors:** 


### ARTICLE VI.

### *The St. Louis Dental Society.*

Dr. W. N. Morrison—I desire to occupy the attention of the Society with the history of a case, which presents some points of interest to me, and upon which I should be pleased to hear the opinion of the members present. Some ten years since a lad about fifteen years of age called to have his teeth put in order. Upon examination I found them in a very bad condition, mainly from neglect. Three of the teeth, one of which I have here, were badly decayed, involving the pulps. I removed the pulps of these teeth, sixth year molars, and filled the roots with gold wire, the crowns with gold, also filled other teeth which needed attention. After the space of eight years my patient returned, complaining of severe pain in this tooth, (the left anterior superior molar.) I found the parts immediately surrounding it turgid and red; tooth elongated, with slight tenderness on percussion; lanced the gums freely, and applied creosote and iodine; suppuration supervened and passed off around the palatine border of the tooth, after which the parts regained their normal condition. A few weeks since my patient again returned, complaining of this old offender. Upon examination I found that from neglect the tooth had been allowed to decay on its mesial surface, reaching the crown filling, but it had not gone far enough to destroy the filling or be the cause of the present trouble. As my patient would not hear to any mode of treatment for relief, except that of extraction, I removed it. I found the buccal roots apparently healthy, the palatine root exostosed and slightly discolored. The alveoli surrounding the tooth appeared to be in a healthy condition; the remaining teeth in good condition, and have never given any trouble since being filled; patient healthy. Why did this tooth, which received the same treatment as the other two which were similarly affected at the same time, give trouble, while they remained quiet. What was the cause of the pain in this case?

Dr. Forbes—The palatine root is exostosed; this exostosis is, to my mind, the key to all the trouble in this case. The irritation about the point of this root, the result of the death of the nerve, owing to the health of the patient, resulted in a new growth being set up, which was so gradual as to cause no pain for ten years, or until it became so large as to impinge upon the surrounding tissues. Had the patient been sickly, the result would have probably been the reverse of exostosis—atrophy.

Dr. Chase—It is not unusual for a tooth treated in this manner to remain quiet for few years, and then give trouble. A small portion of the nerve tissue was probably left in the nerve canal, which in time was decomposed. The discoloration of this palatine root is undoubtedly due to carbon, the result of decomposition. This too might be the source or cause of irritation in the parts surrounding this root, and this irritation might result in the absorption of the root, or in a new growth, exostosis. This exostosis did not cause pain. It is difficult to say what the cause was in this case. We often find cases of teeth giving trouble which to all appearances are perfect and healthy. Nerves die and suppurate without any apparent cause. The presence of a plug will sometimes cause absorption of the dentine, exposing the pulp underneath it. Have seen cases where teeth have been plugged, which in time gave pain, and on removing the filling found the nerve exposed. Have seen other cases where the nerve was exposed when the filling was put in, and on removing the filling sometime afterwards, found the nerve covered with a new deposit. Why this difference we cannot tell.

Dr. Forbes—The gentleman says he cannot assign any reason for absorption taking place in one case and a new deposit in another. I believe that the health of the patient has much to do with the results. With a certain degree of inflammation in a part, with healthy tissues surrounding it, we shall have exostosis, with little or no apparent disturbance attending its formation till it impinges upon the membranes surrounding it—then we may have acute pain.

Dr. Chase—I have seen in unhealthy patients, cases of exostosis. When pus is formed, and a rapid breaking down of tissue takes place, even in healthy patients, we may have both conditions in the same patient. Decalcification of the dentine under filling is a pathological condition; decalcification of the roots of the deciduous teeth, as it occurs in accordance with the laws of nature, is a physiological process.—*Missouri Dental Journal.*

